# A systematic review of the role of community pharmacists in the prevention and control of cardiovascular diseases: the perceptions of patients

**DOI:** 10.1186/s13643-023-02338-7

**Published:** 2023-09-14

**Authors:** Nthabiseng Florina Motlohi, Ebenezer Wiafe, Kofi Boamah Mensah, Neelaveni Padayachee, Ruwayda Petrus, Varsha Bangalee

**Affiliations:** 1 Discipline of Pharmaceutical Sciences, University of KwaZulu-Natal, Durban, South Africa; 2 Ho Teaching Hospital, Ho, Ghana; 3 Faculty of Pharmacy and Pharmaceutical Sciences, Kwame Nkrumah University of Science and Technology, Kumasi, Ghana; 4 Department of Pharmacy and Pharmacology, University of Witwatersrand, Johannesburg, South Africa; 5 Discipline of Psychology, University of KwaZulu-Natal, Durban, South Africa

**Keywords:** Cardiovascular diseases, Role, Community pharmacist, Prevention and control, Patient, Perceptions

## Abstract

**Background:**

Cardiovascular diseases are a leading cause of mortality globally. The modifiable risk factors can be measured and identified early at primary healthcare facilities. Community pharmacists present an opportunity for improved management of cardiovascular diseases and health outcomes. The systematic review aims to identify the roles of community pharmacists in preventing and controlling cardiovascular diseases and patients’ perceptions towards such functions.

**Methods:**

A systematic review of the literature was conducted using the Preferred Reporting Items for Systematic Reviews and Meta-Analysis (PRISMA) guidelines. The team searched MEDLINE, CINAHL via EBSCOhost, and Web of Science from January 2001 to December 2021 with a focus on studies reporting the role of community pharmacists in preventing and controlling cardiovascular diseases, and patients’ perceptions of such roles. Search terms included were ‘‘interventions,’’ ‘‘community pharmacists,’’ ‘‘patients,’’ ‘‘cardiovascular diseases,’’ ‘‘risk factors,’’ and “perceptions”. The quality of studies was appraised using the Joanne Briggs Institute checklist.

**Results:**

A total of 45 studies met the inclusion criteria: 35 (78%) and 10 (22%) reported community pharmacists’ preventive and control roles, respectively. Generally, drug therapy monitoring, medicine and lifestyle counselling, and health education were most common roles, with pharmacist-initiated prescribing and social support least common. A total of 11 (24%) studies reported patients’ perceptions of community pharmacists’ contribution in preventing (73%, *n* = 8) and controlling (27%, *n* = 3) cardiovascular diseases. Patients were satisfied with community pharmacists’ services in 10 of 11 studies.

**Conclusions:**

The findings highlight community pharmacists’ capability of providing primary healthcare services in preventing and controlling cardiovascular diseases and provide evidence for their inclusion in primary healthcare frameworks. Future research should assess the effectiveness of these roles and provide a comprehensive evaluation of clinical, humanistic, and economic outcomes.

**Systematic review registration:**

Open Science Framework (OSF) registration https://doi.org/10.17605/OSF.IO/WGFXT.

**Supplementary Information:**

The online version contains supplementary material available at 10.1186/s13643-023-02338-7.

## Background

Cardiovascular diseases (CVDs) are an umbrella term used to describe disorders of the heart and blood vessels such as coronary heart disease, cerebrovascular diseases, peripheral arterial diseases, rheumatic heart disease, congenital heart disease, deep vein thrombosis, and pulmonary embolism [1]. CVDs are a leading cause of mortality globally. Approximately 18 million deaths occur annually due to CVDs globally [2, 3]. Strikingly, 33% of affected populations were below 70 years, thus imposing suffering and economic difficulties, particularly in low- and middle-income countries (LMICs), which carry over 75% (*n* = 13 million) of CVD-related global mortality [2, 3]. Nonetheless, the impact of CVDs can be minimized by addressing identifiable and modifiable behavioral and physiological risk factors such as the use of tobacco, consumption of an unhealthy diet, overuse of alcohol, inadequate physical activity, hypertension, dyslipidemia, and diabetes [3, 4]. The modifiable behavioral and physiological risk factors can be measured and identified early at primary healthcare (PHC) facilities for early management and improved outcomes.

Community pharmacies are an essential part of PHC. Community pharmacies are located in the communities closer to the users and are the first point of contact for some. They provide expedited services compared with other PHC facilities, such as outpatient hospital clinics, and offer convenience to the users [5, 6]. Community pharmacists thus present an opportunity for improving CVDs’ management and achieving favorable health outcomes. By being located in the community, community pharmacies become easily accessible to a wide range of populations, including hard-to-reach populations, minority groups, and disadvantaged communities that lack the resources to visit other healthcare facilities [5, 7, 8]. They are staffed with community pharmacists who are knowledgeable and skilled in primary healthcare delivery [7]. The public perceives community pharmacists as medication experts with effective communication skills at all levels of society [9, 10]. In LMICs with poor healthcare resources, increasing CVD morbidities and mortalities add a significant strain on healthcare systems and contribute to poor health outcomes [3]. Community pharmacists provide an opportunity to deliver public health interventions for improved CVD prevention and control at a PHC level.

The role of community pharmacists has increasingly grown from being medicine dispensers to becoming crucial role players in disease prevention and control. Community pharmacists can provide effective population-based and individualized PHC services with measurable outcomes [11]. Over the past decade, community pharmacists have shifted their professional role from being task-oriented to dispensing medicines to becoming an integral component in the management of diseases, providing health promotion services that are patient-centered [11–16]. Additionally, the quality of services provided by community pharmacists is evaluated based on the latest model (ECHO) of outcome that adds humanistic (patient-centered outcomes including patient satisfaction, quality of life) and economic outcomes (cost implications) to the traditional clinical outcome (events that occur following disease occurrence or therapy) model [17]. According to Barry and Hughes [17], healthcare decisions about a patient were guided merely by clinical indicators such as blood pressure and blood sugar measurements, and clinical outcomes such as hospitalization and death. The ECHO model provides a comprehensive evaluation of quality care that can be used in decision-making to guide the adoption of alternative treatment models [17].

Previous reviews have reported the role of community pharmacists in the management of CVDs [18–23]. However, they did not explore patients’ perceptions and were limited to a single risk factor or either primary/secondary prevention of CVDs. Furthermore, the studies were either not focused on community pharmacy settings or are now outdated [18, 19, 21–24]. Thus, this study aimed to systematically review the literature to explore the roles of community pharmacists in the prevention and control of CVDs, and the perceptions of patients towards such roles. Specifically, the study seeks to answer the following research questions:

- What are the roles of community pharmacists in the prevention of cardiovascular diseases?
- What contributions do community pharmacists make in the control of cardiovascular diseases?
- What are the perceptions of patients concerning the contributions of community pharmacists in the prevention and control of cardiovascular diseases?

The review provides current evidence of community pharmacists’ evolving roles in preventing and controlling CVDs, and patients’ perceptions towards such functions, in a community pharmacy setting. For the current study, the preventive roles were community pharmacist’s services for patients with reported CVDs risk factors such as hypertension, diabetes, and dyslipidemia but without established CVDs. The control roles (contributions) were community pharmacist’s services for patients with reported established CVDs.

## Methods

### Search strategy and documentation of results

A systematic review of the literature was conducted in January 2022 using the Preferred Reporting Items for Systematic Reviews and Meta-analysis (PRISMA) guidelines, with a focus on studies published between January 01, 2001, and December 31, 2021 [25]. The team searched MEDLINE, CINAHL via EBSCOhost, and Web of Science electronic databases using search terms such as ‘‘interventions,’’ ‘‘community pharmacists,’’ ‘‘patients,’’ ‘‘CVDs,’’ ‘‘CVD risk factors,’’ and “perceptions” (see Additional file 1). NFM designed and finalized the search strategy with documentation provided in Additional file 1. Boolean operators such as “and” and “or” were used to expand the search strategy for optimal results focused on the specific research questions (see Additional file 1). The search strategy was peer-reviewed by two co-authors (EW and VB). The search strategy was run to retrieve relevant citations, which were then exported to the EndNote 20 reference management software package [26].

### Inclusion criteria

The following criteria formed the basis for the inclusion of studies:

- Studies published from January 01, 2001, to December 31, 2021.
- Primary studies with no restrictions on study designs.
- Studies that recruited patients aged 18 years and above with established CVDs and/or CVD risk factors.
- Studies with a community pharmacy setting.
- Studies focused on the role of community pharmacists in preventing and controlling CVDs (primary outcome) and/or patients’ perceptions towards such roles (secondary outcome).
- Studies in the English language.

### Exclusion criteria

The following formed the basis for the exclusion of studies:

- Book chapters, reviews, commentaries, letters to the editor, conference papers, dissertations, and thesis.
- Studies that involved a multidisciplinary team of other healthcare professionals in which the role of community pharmacists was not distinctively described.
- Studies not answering the research questions.
- Studies that were exclusively conducted in hospitals and clinics.

### Data screening and extraction

Titles and abstracts were screened against the inclusion criteria by NFM and EW. Firstly, full articles were retrieved from Google Scholar and through the University of KwaZulu-Natal interlibrary loans for studies that met the inclusion criteria or uncertain titles and abstracts. The full articles were further screened against the inclusion criteria. Finally, a manual reference list screening of eligible studies was performed to identify relevant articles. Data extraction and capturing of data extracts were independently done by two authors (NFM and EW). Any deviations were discussed and settled by KBM, NP, RP, and VB. Data extracts were entered into a customized matrix, comprising details not limited to the authors of included articles, the date of publication, the country where the study was conducted, and the study design (Table 1).

**Table 1:** Characteristics of included studies, findings, and outcomes

**Author (year), country of study**	**Study design, sampling technique**	**Interest population, (sample size)**	**CVDs, co-morbidities**	**Community pharmacists’ role**		**Patients’ perceptions**	**Strengths and limitations**	**Type of outcome, (result)**
				**Prevention of CVDs**	**Control of CVDs**			
Aguwa et al. (2008), Nigeria [27]	Crossover non-randomized, purposive	Hypertensive patients (40)	“Missing,” hypertension/diabetes	Lifestyle counseling Blood pressure (BP) self-care management Smoking cessation Adherence support Hypertension education	“Missing”	“Missing”	**Strengths** Subjects were their own control Pharmacists received program training. **Limitation** Purposive sampling Male-dominated sample (75%) Patient self-reported data	Clinical & humanistic (favorable)
Ali et al. (2003), Canada [28]	Before-after uncontrolled, purposive	Dyslipidemia patients (149)	“Missing,” dyslipidemia	Health education Lifestyle counseling Available therapies Regular follow-up	“Missing”	The program was perceived as satisfactory and patients were willing to pay for the program	**Strengths** Pharmacists received program training **Limitations** No comparator Purposive sampling No randomisation	Clinical & humanistic (favorable)
Ali et al. (2012), UK (UK) [29]	Randomized controlled trial (RCT), random	Diabetes (type 2) patients (48)	“Missing,” diabetes	Medicine use review Lifestyle counseling Referrals Regular follow-up & monitoring Diabetes education	“Missing”	Patients perceived their knowledge of diabetes and health status were improved following education program	**Strengths** Allocation concealment Computer-generated random list Low inter-rater Pharmacists received program training High retention rate **Limitations** Possible group contamination Caucasian-dominated sample Smaller sample size	Clinical & humanistic (favorable)
Al Hamarney et al. (2012), Canada [30]	Cross-sectional, purposive	Diabetic patients (200)	“Missing,” diabetes	Detection of poorly controlled diabetic patients	“Missing”	“Missing”	**Strengths** Subjects identified through medical records **Limitations** Purposive sampling Elderly dominated sample	Not applicable
Al Hamarney et al. (2013), Canada [31]	Before-after uncontrolled, purposive	Patients with poorly controlled diabetes (type 2) (100)	“Missing,” hypertension/diabetes/dyslipidemia	Medication use counseling Self-care management Pharmacist-initiated insulin prescription	“Missing”	The patients perceived the community treatment as satisfactory	**Strengths** Pharmacists received program training Intention-to-treat analysis **Limitations** White-dominated sample No comparator Purposive sampling	Clinical & humanistic (favorable)
Al Hamarneh et al. (2017), Canada [32]	RCT, random	Diabetic/CVD risk patients (573)	Atherosclerotic vascular disease Heart failure Peripheral arterial disease Atrial fibrillation, hypertension/diabetes/dyslipidemia/chronic kidney disease (CKD)	Pharmacotherapy management (medicine therapy management) CVD risk screening CVD education Referrals Treatment recommendation Pharmacist-initiated prescription Regular follow-up & monitoring	CVD risk screening CVD education Referrals Treatment recommendation Prescription initiation Regular follow-up & monitoring	‘‘Missing’’	**Strengths** Allocation concealment Intention-to-treat analysis Control & treatment groups comparable at baseline Larger sample size **Limitations** No blinding Patient self-reported data	Clinical (favorable)
Al Hamarneh et al. (2018), Canada [33]	Cross sectional interviews, purposive	CVD risk patients (14)	“Missing,” hypertension/diabetes/CKD.	CVD risk screening	“Missing”	Community pharmacists were compassionate, collaborators, & articulate Patients were highly satisfied with pharmacist care	**Strengths** Data analysed by 3 independent reviewers **Limitations** Purposive sampling Interviews/opinions (information bias) Subjects selected by pharmacists (selection bias).	Humanistic (favorable)
Aslani et al. (2011), Australia [34]	Cluster randomized trials (CRT), random	Dyslipidemia patients (142)	“Missing,” dyslipidemia	Adherence support Regular follow-up & monitoring	‘‘Missing’’	‘‘Missing’’	**Strengths** Pharmacists received program training Control & treatment groups comparable at baseline Minimal group contamination (cluster sampling) **Limitations** Findings limited to pharmacy users Smaller sample size Higher dropout (32%) Pharmacists compensated	Clinical (favorable)
Blackburn et al. (2016), Canada [35]	CRT, random	Statin users (1906)	“Missing,” dyslipidemia	Adherence support	‘‘Missing’’	‘‘Missing’’	**Strengths** Allocation concealment Randomization Pharmacists received program training Control & treatment groups comparable at baseline Minimal group contamination (cluster sampling) Broader representation of pharmacy type **Limitations** Findings to limited new statin users One state	Humanistic (unfavorable)
Boardman & Avery (2014), UK [36]	Cross-sectional, purposive	CVD risk patients (281)	“Missing,” hypertension/diabetes/dyslipidemia	Lifestyle counseling Smoking cessation Regular follow-up & monitoring	“Missing”	“Missing”	**Strengths** Broader pharmacy types representation Pharmacist & research assistants received program training **Limitations** The program differed across pharmacies Purposive sampling No comparator White & female-dominated sample	Clinical & humanistic (favorable)
Chabot et al. (2003), Canada [37]	Before-after uncontrolled, purposive	Hypertensive patients (111)	“Missing,” hypertension	Regular follow-up & monitoring Adherence support Treatment recommendations	“Missing”	“Missing”	**Strengths** Blinding of data collectors Pharmacists & research assistants received program training Minimal group contamination (cluster sampling) **Limitations** Pharmacists remunerated Treatment & control groups incomparable at baseline No randomization	Clinical & humanistic (favorable)
Cranor et al. (2003), USA [38]	Before-after uncontrolled, purposive	Diabetic patients (323)	“Missing,” diabetes	Diabetes education Regular follow-up & monitoring Self-care management Adherence support Physical examination Referrals	“Missing”	“Missing”	**Strengths** 5 years of follow-up Intention-to-treat analysis Pharmacists received program training **Limitation** No randomization No comparator Missing data	Clinical, humanistic, & economic (favorable)
Fahs et al. (2018), Lebanon [39]	Longitudinal before-after uncontrolled, convenience	Patients without CVDs (865)	“Missing,” hypertension/diabetes/dyslipidemia	Lifestyle counseling CVD education	‘‘Missing’’	‘‘Missing’’	**Strengths** Rural & urban setting 6 districts represented Standard questionnaire **Limitations** Findings limited to ≥ 45 years Convenience sampling No comparator Patient self-reported data	Clinical & humanistic (favorable)
Fikri-Benbrahim et al. (2013), Spain [40]	Before-after controlled, purposive	Hypertensive patients (209)	“Missing,” hypertension	Adherence support Health education Referrals Home BP device Self-care management DRP identification Regular follow-up & monitoring	“Missing”	“Missing”	**Strengths** Pharmacist received program training Control & treatment groups comparable at baseline **Limitations** Protocol analysis No randomisation Smaller sample size Possible subject contamination No blinding Possible selection bias (more adherent subjects)	Humanistic (favorable)
Fonseca et al. (2021), Portugal [41]	Cross-sectional, convenience	Patients with CVD/risk factors (588)	“Missing,” hypertension/diabetes/dyslipidemia	CVD education CVD risk screening	‘‘Missing’’	‘‘Missing’’	**Strengths** Pharmacist received program training **Limitations** Single centre Convenience sampling No comparator Patient self-reported data	Not applicable
Horgan et al. (2010), UK [42]	Cross-sectional, purposive	Patients with CVD risk factors (1141)	“Missing,” hypertension/diabetes/dyslipidemia	CVD risk screening Referral	“Missing”	“Missing”	**Strengths** Broader pharmacy type representation **Limitations** White dominated sample Findings limited to poor health indicators setting	Not applicable
Hourihan et al. (2003), Australia [43]	Cross-sectional, convenience	Not on dyslipidemia/hypertension treatment (204)	“Missing,” hypertension/dyslipidemia	Health education CVD risk screening Lifestyle counseling Smoking cessation Regular follow-up & monitoring Referrals	“Missing”	Community pharmacist-led healthcare services were convenient	**Strengths** Pharmacists received program training Regular calibration of meters **Limitations** Findings limited to rural setting Convenience sampling. Free service might have encouraged patient participation	Humanistic (favorable)
Hunt et al. (2013), UK [8]	Cross sectional, convenience	Patients without CVDs, (3125)	“Missing”, hypertension/diabetes/dyslipidemia	CVDs risk screening Referral Lifestyle counselling	“Missing”	“Missing”	**Strengths** Balanced gender representation **Limitations** Findings limited to minority groups. Single state Convenience sampling	Not applicable
Jaffray et al. (2007), England [44]	RCT, random	Coronary heart disease (CHD) patients (1614)	Coronary heart disease (CHD), hypertension/diabetes/dyslipidemia	“Missing”	Medication use review Therapy monitoring Medication counseling Lifestyle counseling Smoking cessation Social support Referrals Prescription recommendations	Patients were satisfied with pharmacist care	**Strengths** Outcome assessors blinded Pharmacists received program training Computer-generated randomization Control & treatment groups comparable at baseline **Limitations** Patient self-reported data Participation restricted to pharmacies with consultation rooms	Clinical & economic, (unfavorable), humanistic (favorable)
Jahangard-Rafsanjani et al. (2017), Iran [45]	Cross-sectional, convenience	Subjects with no CVDs or diabetes (287)	“Missing,” hypertension/dyslipidemia	CVD risk screening Lifestyle counseling CVD education. Referrals	‘‘Missing’’	‘‘Missing’’	**Strengths** The use of high precision testing devices **Limitations** Smaller sample size. Single center Urban setting No comparator	Not applicable
John et al. (2006), USA [46]	Before-after uncontrolled, purposive	Individuals with CVD risk factors (58)	“Missing,” hypertension/diabetes/dyslipidemia	CVDs education CVDs risk screening Lifestyle counseling. Smoking cessation DRP identification Regular follow-up & monitoring Treatment recommendations	‘‘Missing’’	‘‘Missing’’	**Strengths** Workplace setting encourages complete follow-up Subjects served as their own controls **Limitations** Rural setting Smaller sample size male-dominated sample No comparator	Clinical (favorable)
Katoue et al. (2013), Kuwait [47]	Cross-sectional, random	Community pharmacists (220)	“Missing,” metabolic syndrome	Screening tests Lifestyle counseling Smoking cessation Adherence support Self-care management Referrals	‘‘Missing’’	‘‘Missing’’	**Strengths** High response rate (97.8%) Bigger sample size Rural & urban setting Questionnaire piloted **Limitations** Questionnaire survey not preferred to explore views	Not applicable
Khettar et al. (2021), France [48]	Cross-sectional, convenience	Community pharmacists (104)	Stroke, “missing”	‘‘Missing’’	Medicine use/management review Lifestyle counselling. Smoking cessation.	‘‘Missing’’	**Strengths** Questionnaire piloted & expert-reviewed **Limitations** Low response rate (1.9%) Youth and male-dominated sample Patient self-reported data Convenience sampling	Not applicable
Krass et al. (2007), Australia [49]	CRT, random	Diabetes (type 2) patients (335)	“Missing,” hypertension/diabetes/dyslipidemia	Adherence support Lifestyle counseling Medicine use review Self-care management DRP identification Referrals Regular follow-up & monitoring	“Missing”	“Missing”	**Strengths** Urban and rural setting Multi-states Minimal group contamination (cluster sampling) Pharmacists received program training Subject eligibility verified through medical records Subjects provided one brand device for self-monitoring **Limitations** Pharmacists remunerated Missing data Significant high drop-out rate in younger participants	Clinical & humanistic (favorable)
Kwint et al. (2012), Netherlands [50]	Cross-sectional, purposive	Patients taking cardiovascular or anti-diabetic drugs (155)	Coronary artery disease (CAD) Cerebral vascular disease Arrhythmia Heart failure, hypertension/diabetes/dyslipidemia/pulmonary disease/artrosis/osteoporosis	“Missing”	DRP identification Home visits Medication reviews Adherence support	“Missing”	**Strengths** Pharmacists received program training Experienced independent program reviewers Independent assessors **Limitations** Findings limited to home dwelling elderly Patient self-report data No comparator Purposive sampling	Not applicable
Marfo & Owusu-Daaku (2017), Ghana [51]	Before-after controlled, purposive	Hypertensive patients, (180)	“Missing,” diabetes	DRP identification Adherence support Medicine use review Lifestyle counseling Health education	“Missing”	Majority of patients were satisfied with community support services	**Strengths** Control & treatment groups comparable at baseline Minimal group contamination (cluster sampling) Pharmacists received program training **Limitations** Pharmacists remunerated Purposive sampling No randomisation Smaller sample size	Clinical & humanistic (favorable)
McNamara et al. (2015), Australia [52]	Before-after uncontrolled, purposive	Patients with hypertension & dyslipidemia, without CVDs/diabetes (70)	“Missing,” hypertension/dyslipidemia	Drug therapy management Adherence support Lifestyle counseling CVD education Regular follow-up & monitoring Treatment recommendations	“Missing”	“Missing”	**Strengths** Pharmacists received program training **Limitations** Female-dominated, rural patients Patient self-reported data No comparator Smaller sample size	Humanistic, (favorable)
Niquille & Bugnon (2010), Switzerland [53]	Cross-sectional, purposive	Patients on cardiovascular drugs (92)	“Missing,” hypertension/diabetes/dyslipidemia	Medication review	“Missing”	“Missing”	**Strengths** Pharmacists received program training **Limitations** Recruitment done by community pharmacists Findings limited to insured participants Smaller sample size Purposive sampling	Clinical, humanistic & economic (favorable)
Okada et al. (2016), Japan [54]	CRT, random	Diabetes patients (163)	“Missing,” diabetes	Lifestyle counseling Diabetes education Self-care management Adherence support Regular follow-up & monitoring.	“Missing”	“Missing”	**Strengths** Blinding of data analysts Allocation concealment Low inter-rater Pharmacists received program training Minimal group contamination (cluster sampling) Randomisation **Limitations** Findings limited to chain pharmacies No blinding Smaller sample size	Clinical & humanistic (favorable)
Okada et al. (2017), Japan [55]	CRT, random	Hypertensive patients (125)	“Missing,” hypertension	Lifestyle counseling Self-care management Regular follow-up & monitoring	“Missing”	“Missing”	**Strengths** Pharmacists received program training Participants received validated BP monitors Minimal group contamination (cluster sampling) Randomization **Limitations** Patient self-reported data Smaller sample size Differences in groups’ baseline data.	Clinical (favourable) & humanistic (unfavorable)
Olenak & Calpin (2010), USA [56]	Cross-sectional, convenience	Subjects without CHD history (239)	“Missing,” metabolic syndrome	CVD risk screening Lifestyle counseling Smoking cessation	“Missing”	Patients perceived community pharmacist’s screening program as satisfactory	**Strengths** Participation not restricted to pharmacy patients Use of point-of-care device **Limitations** Women-dominated sample Patient self-reported data Convenience sampling Single state Free program might have encouraged participation	Clinical & humanistic (favorable)
Oser et al. (2017), USA [57]	Before-after uncontrolled, purposive	Patients on hypertensive medication (534)	“Missing,” hypertension	Adherence support Regular follow-up & monitoring Lifestyle counseling Referrals Medication management	“Missing”	“Missing”	**Strengths** Pharmacists received program training All eligible pharmacies were invited to participate **Limitations** No comparator Rural setting Incentives might have encouraged participation of pharmacists Purposive sampling	Humanistic (favorable)
Peletidi et al. (2019) UK & Greece [58]	Cross sectional interviews, convenience, snowball & random	Community pharmacists (40)	“Missing,” “missing”	Lifestyle counseling Smoking cessation Adherence support Medicine use review (MUR) New medicine service (NMS) CVD screening	“Missing”	“Missing”	**Strengths** Questionnaire piloted & expert-reviewed Congruency between aim and design, data collection & analysis Random sampling (low bias) **Limitations** Findings limited to independent pharmacies Convenience & snowball sampling	Not applicable
Puspitasari et al. (2013), Australia [59]	Cross-sectional interviews, purposive	Community pharmacists (21)	“Missing,” “missing”	“Missing”	Medicine counseling Lifestyle counseling CVD education Medicine use review Patient home visits	“Missing”	**Strengths** Questionnaire-piloted & expert-reviewed Congruency between aim and design, data collection & analysis Rural & urban setting Broader representation of pharmacy types **Limitations** Findings limited to independent pharmacy setting Purposive sampling	Not applicable
Robinson et al. (2010), USA [60]	Before-after controlled, purposive	Patients with uncontrolled hypertension (376)	“Missing,” hypertension	Adherence support DRP identification Hypertension education	“Missing”	“Missing”	**Strengths** Pharmacists received training Control & treatment groups comparable at baseline Patients were identified through prescription databases **Limitations** No randomisation Per protocol analysis Purposive sampling Missing data Findings limited chain pharmacies	Clinical & humanistic (favorable)
Sandhu et al. (2018), Canada [61]	Cross sectional, random	Community pharmacists, (139)	Atrial fibrillation, “missing”	“Missing”	Identification of preventive therapy eligible CVD patients Physician-guided prescribing	“Missing”	**Strengths** Random sampling **Limitations** One city Questionnaire not piloted Smaller sample size	Not applicable
Sia et al. (2020), Malaysia [62]	Cross-sectional, convenience	Community pharmacists (182)	“Missing,” “missing”	“Missing”	CVD screening Lifestyle counseling Smoking cessation	“Missing”	**Strengths** Questionnaire-piloted & expert-reviewed) **Limitations** Urban setting Patient self-reported data Convenience sampling Smaller sample size	Not applicable
Simpson et al. (2004), Canada [63]	RCT, random	Patients with CVDs & risk factors (675)	“Missing,” hypertension/diabetes/dyslipidemia	CVDs risk screening. CVD education Referral Regular follow-up & monitoring	“Missing”	“Missing”	**Strengths** Randomization Control & treatment groups comparable at baseline Pharmacists received program training **Limitations** Patient self-reported data Smaller sample size	Clinical (favorable)
Stewart et al. (2014), Australia [64]	CRT, random	Hypertensive patients (395)	“Missing,” hypertension	Adherence support BP monitor Self-care management Health education DRP identification Home-based therapy review Referrals Refill reminders Regular follow-up & monitoring	“Missing”	“Missing”	**Strengths** Multi-center Urban & rural setting Minimal group contamination (cluster sampling) Pharmacists received training Patients’ data verified through a software Replicate measurements Intention-to-treat analysis Treatment & control groups comparable at baseline **Limitations** Pharmacists remunerated Patient self-reported data No blinding	Clinical & humanistic (favorable)
Thompson et al. (2020), USA [65]	Cross-sectional, convenience	Hypertensive patients, (61)	“Missing,” hypertension	Medication review Lifestyle counseling Self-care management Hypertension education Adherence support	“Missing”	Community pharmacist-led MTM was highly satisfactory	**Strengths** Rural & urban setting Pharmacists received program training **Limitations** Findings limited to insurance members Smaller sample size Convenience sampling No comparator	Humanistic (favorable)
Tsuyuki et al. (2002), Canada [66]	RCT, random	Patients with CVDs/CVDs risk factors (675)	Atherosclerotic vascular disease, diabetes	“Missing”	Point-of-care testing CVD education Referrals Follow-ups Adherence support	Community pharmacist-led program was satisfactory	**Strengths** Allocation concealment Intention-to-treat analysis Treatment & control groups comparable at baseline **Limitations** Patients selected by pharmacists Limited findings limited to pharmacy users Smaller sample size	Clinical & humanistic (favorable)
Tsuyuki et al. (2004), Canada [67]	Before-after uncontrolled, random	Patients with CVD risk factors/CVD risk factors (419)	Atherosclerotic vascular disease, hypertension/ diabetes/dyslipidemia	“Missing”	Lifestyle counselling Adherence support Health education DRP identification	“Missing”	**Strengths** Pharmacists received program training Replicate measurements High precision device Multi-center Randomization **Limitations** Patients selected by pharmacists No comparator	Clinical & humanistic (favorable)
Tsuyuki et al. (2016), Canada [68]	RCT, random	CVD/CVD risk factors (723)	Atherosclerotic vascular disease Heart failure Atrial fibrillation, hypertension/dyslipidemia/diabetes/CKD	CVD risk screening CVD education Treatment recommendations Smoking cessation Regular follow-up & monitoring	CVD education Treatment recommendations Smoking cessation Regular follow-up & monitoring	“Missing”	**Strengths** Allocation concealment Computer-generated randomization Pharmacists received program training Treatment & control groups comparable at baseline Intention-to-treat analysis **Limitations** Shorter follow-up period (3 months) Single state Patient self-reported data)	Clinical & humanistic (favorable)
van Geffen et al. (2011), Netherlands [69]	Cross-sectional, convenience & random	Patients on CVD treatment (1546)	“Missing,” hypertension/diabetes/dyslipidemia	Medicines counseling advice	“Missing”	Patients were dissatisfied with & perceived community pharmacists as incapable to provide sufficient medication information	**Strengths** Urban & rural setting Random sampling **Limitations** Elderly-dominated sample Findings limited to networked pharmacies Possible information bias (patients’ views)	Humanistic (unfavorable)
Zillich et al., (2005), USA [70]	CRT, random	Hypertensive patients with uncontrolled BP (125)	“Missing,” hypertension	Hypertension education Self-care management Lifestyle counseling Medication counseling Adherence support Referral Home BP device Regular follow-up & monitoring	“Missing”	“Missing”	**Strengths** Pharmacists received program training Control & treatment groups comparable at baseline Minimal group contamination (cluster sampling) **Limitations** Findings limited to networked pharmacists No randomization Pharmacists remunerated	Clinical & humanistic (favorable)

### Quality assessment of eligible studies

The quality of eligible studies was assessed using critical appraisal tools by the Joanna Briggs Institute (JBI) in Australia [71]. The JBI provides quality assessment tools for various study designs and is suitable for systematic reviews that combine different study designs [72]. Appropriate critical appraisal tools were used for randomized controlled trials (RCTs) and cluster randomized trials (CRTs), quasi-experimental trials, prevalence, and qualitative studies [73–75]. Quality assessment was performed independently by two authors (NFM and EW). A point (one) was allocated to a “yes” response if the study met quality requirements based on the criteria of a critical appraisal tool.

### Data analysis and synthesis

The characteristics of the included studies and study findings were summarized and computed as sum and percentages using Microsoft Excel 2013 version [76]. The outcome of each included study was classified as clinical, economic, and/or humanistic according to the ECHO model [17]. For the purposes of this study, clinical outcomes cover clinical indicators such as blood pressure, blood sugar, serum level, and inpatient hospitalization and death. A meta-analysis was not performed due to (1) the aim of the study and (2) the different designs of the included studies (heterogeneity) which did not support meta-analysis [77].

## Results

### Description of the studies

**Fig. 1:**
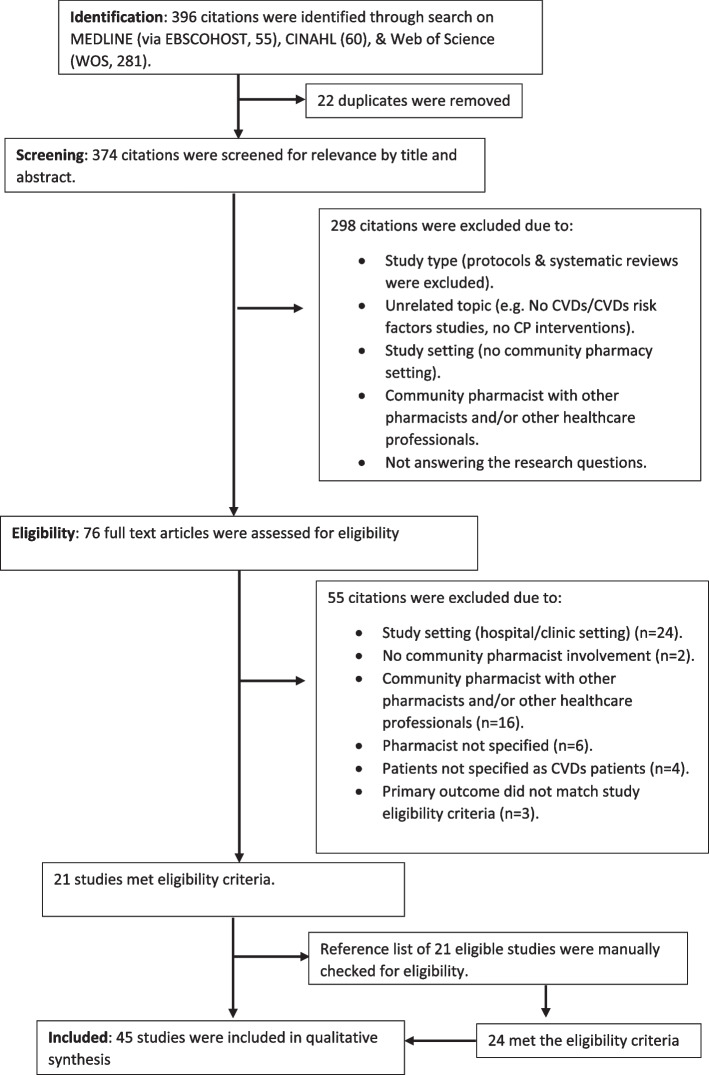
Flow chart outlining literature search and screening of studies for eligibility

The initial online literature search resulted in 396 citations from MEDLINE (55), CINAHL (60), and Web of Science (281). A flow diagram illustrating the steps followed in screening citations and identifying studies that met the eligibility criteria is presented in Fig. 1. A total of 45 studies were finally included in the review. The studies were published between 2002 and 2021, with the majority (73%, *n* = 33) published between 2010 and 2021 (Table 1). The collection of studies represented 18 countries. Most studies occurred in high-income countries (HICs) (89%, *n* = 40) whilst 11% of the studies were conducted in LMICs (*n* = 4) and upper-middle income countries (UMICs) (*n* = 1). In HICs, Canada (*n* = 2) and the USA (*n* = 7) contributed most papers whilst in LMICs, each country had 1 eligible study. The review included only one multinational study, the UK and Greece [58]. The study designs were observational studies (42%, *n* = 19), randomized controlled trials (29%, *n* = 13), and quasi-experimental (29%, *n* = 13) with sample sizes ranging between 14 and 3125 participants (Table 1). The sampling techniques used were purposive/convenience (60%, *n* = 27), random (36%, *n* = 16), and a combination of different techniques (4%, *n* = 2).

### Methodological quality assessment of the included studies

The included studies were classified according to their study designs (Table 1) and appraised using an appropriate JBI critical appraisal tool. The methodological quality of the studies varied with study designs. The results of the methodological quality assessment are summarised as the studies’ strengths and limitations in Table 1. The scorings could be found in Additional file 2. For the most part, the intervention and control groups had comparable characteristics at baseline in RCTs and CRTs (Table 1), thus minimizing selection bias that could potentially overestimate or underestimate effect size. Similarly, the study subjects did not receive treatment other than the controlled intervention of interest. This suggests that the effect could strongly be attributed to the intervention. Contrarily, most studies did not blind neither the participants to treatment assignment nor those assigning treatment to participants mainly due to the nature of the interventions. It was impossible to blind the participants. This could have encouraged participants to react or behave differently, or those assigning treatment to treat participants differently from the control group, thus overestimating or underestimating the study outcomes.

Regarding prevalence studies, most studies described participants and study settings in details. This could aid an informed judgment regarding the applicability of the study findings. On the other hand, most studies used convenience/purposive sampling to select study participants, thus subjecting the results to selection bias and consequently lack of generalization. Additionally, there was a low response rate and most studies lacked clear reporting on reasons for unresponsiveness. The majority of quasi-experimental designs met the quality criteria. However, the absence of a control group possibly underestimated the validity of causal relationships between the effect and the intervention. Generally, all studies had a potential for bias in their design, conduct, and analysis. Therefore, readers should interpret the review’s findings with caution.

### The role of the community pharmacist in the prevention and control of CVDs

The role of community pharmacists in CVD prevention and control can be broadly classified into two categories namely primary (prevention of CVDs by addressing modifiable risk factors) and secondary (prevention of recurrent events in people with established CVDs) prevention of CVDs [3]. Therefore, preventive roles pertain to primary prevention, while control roles pertain to secondary prevention.

#### Preventive roles

A total of 35 out of 45 studies (78%) reported preventive roles. The roles were categorized into 11 themes namely, medicine and lifestyle counseling (66%, [23/35]), health education (63%, [22/35]), regular consultations and therapeutic monitoring (60%, [21/35]), adherence support (57%, [20/35]), drug therapy review (43%, [15/35]), referrals to physicians (40%, [14/35]), CVD risk screening (37%, [13/35]), self-care management (29%, [10/35]), smoking cessation (23%, [8/35]), treatment recommendations (14%, [5/35]), and pharmacist-initiated prescribing (6%, [2/35]). The least common preventive role was pharmacist-initiated prescribing (*n* = 2) reported in Canada [31, 32] with favorable clinical outcomes. For instance, approximately 1.8% (CI 95% 1.4–2, *P* < 0.0001) change in glycaemic control and 4.1 mmol/L (CI 95% 3.3–5, *P* = 0.007) decrease in fasting blood glucose were achieved in 51% of the enrolled patients following initiation of pharmacist-prescribed insulin [31].

#### Control of CVDs

Generally, fewer studies reported community pharmacists’ roles in the control of CVDs (22%, *n* = 10). Most included studies reported at least two control roles each. The roles were categorized into 12 themes, namely, drug therapy review (50%, [5/10]), medicine and lifestyle counseling (50%, [5/10]), health education (50%, [5/10]), smoking cessation (40%, [4/10]), referrals to the physicians (30%, [3/10]), regular consultations and therapeutic monitoring (30%, [3/10]), adherence support (30%, [3/10]), treatment recommendations (30%, [3/10]), CVD risk screening (20%, [2/10]), pharmacist-initiated prescribing (20%, [2/10]), identification of preventive therapy eligible CVD patients (10%, [1/10]), and social support (10%, [1/10]) (Table 1). Most of the CVD control roles were identified under the CVD preventive roles except two: identification of preventive therapy-eligible CVD patients and social support assessment. A 27% of the studies explored community pharmacists’ perceptions on their role in the management of CVDs and consequently reported no outcomes.

### Patients’ perceptions of community pharmacist’s role in the prevention and control of CVDs

A total of 11 (24%) studies reported patients’ perceptions of community pharmacists’ role in the prevention (73%, *n* = 8) and control (27%, *n* = 3) of CVDs (Table 1). CVD patients were dissatisfied with medicine counseling services provided by community pharmacists in 1 of 11 studies [69]. For the most part, patients perceived community pharmacists’ role (medicine and lifestyle counseling, medicine therapy management, screening services, disease education, prescribing) as satisfactory [29, 31, 43, 44, 51, 66] and showed a willingness to use services in the future. Similarly, community pharmacists were described as empathetic, collaborative, and communicative, and patients found it convenient to consult a community pharmacist.

## Discussion

To the best of the authors’ knowledge, this is the first systematic review that focuses on the role of community pharmacists in preventing and controlling CVDs, and patients’ perceptions of such roles. Community pharmacists’ role in preventing and controlling diseases is evolving and has been complemented by an increase in research. This is supported by the increasing number of publications (73%) on the role of the community pharmacist in the management of CVDs over the past decade, adding more insights to the body of knowledge. The review identified drug therapy review, medicine and lifestyle counseling, health education, smoking cessation, referrals to the physician, regular consultations and therapeutic monitoring, adherence support, treatment recommendations, CVD risk screening, pharmacist-initiated prescribing, identification of preventive therapy eligible CVDs patients, and social support as community pharmacist’ roles in the prevention and/or control of CVDs. Although less reported, CVD patients perceived community pharmacists’ health promotion roles as satisfactory and showed a willingness to use services in the future [29, 31, 43, 44, 51, 66]. Therefore, the review presents background information that supports community pharmacists’ involvement in the primary and secondary prevention of CVDs and their potential to contribute towards desired health outcomes.

The review unearthed contributions of community pharmacists that can potentially improve clinical, humanistic, and economic outcomes in CVD patients. Findings of a non-randomized crossover study conducted in Nigeria showed improved blood pressure in hypertensive patients following a lifestyle counseling and adherence support [27]. Patients’ adherence to drugs and diet recommendations, self-care management, and quality of life also improved. In addition to improved blood pressure, Boardman and Avery [36] reported an improvement in weight control following a 6-month weight management support program [36]. Similar blood pressure improvements were supported by Fahs and Hallit [39] with an improved lipid profile and CVD knowledge by patients following lifestyle counseling and CVD education [39]. Moreover, findings by Tsuyuki and Al Hamarneh [68] demonstrated improvement in cholesterol, systolic blood pressure, glycosylated hemoglobin, and smoking cessation [68]. Along with clinical and humanistic outcomes, community pharmacist-led health promotion programs showed a decrease in mean total direct medical costs after a 9-month follow-up on diabetic patients [38].

The results build on previous findings in which community pharmacist-led health promotion activities showed a considerable benefit in improving CVD risk factors [18, 23, 78]. However, the pharmacist’s role in facilitating patient group discussions was not part of our findings [78]. Correspondingly, pharmacist-initiated prescribing and social support were unique to this review and least frequently reported [31, 32, 44]. The clinical outcome for pharmacist-initiated prescribing was a substantial reduction in CV risk contributed by improved blood pressure, blood glucose, and cholesterol measurements and tobacco use over a period of 3 months. Interestingly, the findings were comparable to past physician-led investigations [31].

Although the majority of the contributions reported favorable outcomes, undesirable health outcomes were observed in some studies. For instance, a CRT concluded that medication adherence support did not improve adherence in patients on statin therapy in Canada [35]. Likewise, in another CRT conducted in Japan, a lifestyle program did not improve the quality of life and knowledge about lifestyle in hypertensive patients, though there was a significant change in blood pressure between the intervention and comparison groups [55]. Additionally, an RCT conducted in England revealed that pharmacist health promotion services were more expensive compared with standard care [44]. Generally, there was heterogeneity in the conduct of studies in various settings. For instance, the variability was observed in study designs and settings, length of follow-up, presence/absence of comparator group, subject recruitment, inconsistency in program implementation, and lack of standardization in outcome measures across study sites (Table 1). These variabilities could potentially overestimate/underestimate the outcomes. Therefore, future studies should focus on developing standardized guidelines for community pharmacy implementation, monitoring, and evaluation of community pharmacist-led interventions towards improved prevention and control of CVDs.

Generally, the types of roles have remained essentially the same in the past two decades [18, 21–23, 78]. Nonetheless, social support assessment, pharmacist-initiated prescribing, and identification of CVD preventive therapy-eligible patients were uncommon and restricted in the HICs [31, 32, 44, 61]. This highlights an opportunity for community pharmacists to expand their provision of services to CVDs particularly in LMICs which carry the highest CVD mortality globally [3]. Most CVD control roles were identified under the CVD preventive roles except for two: identification of preventive therapy-eligible CVD patients and social support assessment. Through the identification of patients that are eligible for preventive therapy, community pharmacists are well positioned to recommend treatment to the physicians and facilitate timely initiation of treatment to patients at risk of CVD events such as stroke. The social support assessment was a component of a medicine management service package provided by community pharmacists to patients with established CVDs in England (Table 1). The overall cost of the service was higher in the intervention group compared with the control group, contributing to unfavorable economic outcomes. Nonetheless, overall patients’ satisfaction with community pharmacists’ services significantly improved.

There were fewer (22%) community pharmacists’ roles in the control compared with their contributions to the prevention of CVDs. This could be due to publication bias resulting from selective reporting [79]. Pharmaceutical care for patients with established CVDs is considered routine work for most pharmacists compared with patients with no disease. Therefore, it is possible that the results of the investigations were not considered for publication. Publication bias is common in healthcare research and one of the contributors to incomplete information available in healthcare decision-making [79].

The success of a pharmaceutical care intervention is weighed on the ECHO model [17]. Patients’ perceptions are an important element of humanistic outcomes and contribute massively towards the success of healthcare programs. According to the Theory of Planned Behavior, patients’ behavioral beliefs and attitude guide their intention to utilize healthcare services that contributes towards positive or negative outcomes [80]. If patients have concerns about a healthcare service, and those concerns are not addressed, they might not utilize such services. This underscores the importance of a more inclusive approach that takes into consideration all key stakeholders in healthcare systems, including patients, for better outcomes. Only 24% of the studies reported patients’ views toward the role of community pharmacists and their intention to utilize such services. Future studies to adopt the ECHO model of outcomes comprehensively to guide the development of frameworks that incorporate community pharmacists in the primary healthcare models. Despite that, patients perceived community pharmacists’ roles mainly as satisfactory and convenient. These results provide evidence of community pharmacists’ potential to deliver patient-centered services to CVD patients.

The findings of the review should be read in light of the study’s limitations. Firstly, studies published in other languages other than English were excluded. These studies could potentially add a plethora of information regarding the role of community pharmacists in preventing and controlling CVDs, and patients’ perceptions thereof. Secondly, the majority of the studies were conducted in HICs (89%), leaving a gap in the body of knowledge regarding the role of community pharmacists and the application of the results in preventing and controlling CVDs in LMICs. Moreover, most studies were uncontrolled (60%) and used non-probability sampling techniques, suggesting overestimation or underestimation, and lack of representation of the findings. Among the studies that had a control group (*n* = 19), 4 studies used a non-randomized approach to select participants (Table 1), subjecting the results to possible selection bias. Participants were selected through community pharmacy users’ databases, referred by their physicians, and judged as eligible by their pharmacists, while others volunteered to participate after reading a study advert placed at the pharmacies. It was possible that patients who self-referred themselves had effective self-management and were more motivated than those who did not participate (volunteer bias). To improve the validity of the outcomes of community pharmacist services, and to understand their effectiveness, study designs that reduce bias to research findings such as randomized controlled study designs should be considered for future research (Wagoner, 2004, as cited in [81]). Furthermore, the authors used their judgment to score the quality of the studies as there was no standard to benchmark against [71]. Therefore, caution should be exercised in the interpretation of quality scores. Nevertheless, the authors are confident that the results are less subjective as two independent people agreed on the quality scores. The review provides a piece of global evidence on the roles of community pharmacists in preventing and controlling CVDs, and the perceptions of patients towards such roles.

## Conclusion

In summary, the role of community pharmacists is evolving and becoming more patient-centered. Community pharmacists’ roles in CVD care were largely preventive and mainly included medicine and lifestyle counseling, health education, regular consultations and therapeutic monitoring, and adherence support. Patients’ perceptions were less investigated, highlighting the need for future research to include this element of the ECHO model. Generally, the findings of this review underlined the potential of community pharmacists as important healthcare professionals who can provide primary healthcare care services in the prevention and control of CVDs. The roles might contribute immensely to the successful implementation of healthcare programs aimed at reducing the incidence and impact of CVDs. Future research to explore the role of community pharmacists in other countries, particularly the LMICs, evaluate the clinical, humanistic, and economic outcomes, and determine the effectiveness of the interventions using robust controlled study designs.

### Supplementary Information

**Additional file 1.** Proposed databases, search strategies and results. Medline via EBSCOhost.

**Additional file 2. **

## Data Availability

None.

## Abbreviations

CVDs: Cardiovascular diseases
LMICs: Low- and middle-income countries
PHC: Primary healthcare
ECHO: Clinical, humanistic, and economic outcomes
PRISMA: Preferred Reporting Items for Systematic Reviews and Meta-Analysis
JBI: Joanna Briggs Institute
RCTs: Randomized controlled trials
UMICs: Upper-middle income countries
HICs: High-income countries
US: United States
UK: United Kingdom
BP: Blood pressure
CKD: Chronic kidney disease
CRT: Cluster randomized trial
DRP: Drug-related problems
CHD: Coronary heart disease
CAD: Coronary artery disease
MUR: Medicine use review
NMS: New medicine service
