# Supplementary material for: A systematic review of the role of community pharmacists in the prevention and control of cardiovascular diseases: the perceptions of patients
Source: Syst Rev. 2023 Sep 14;12:160. doi: 10.1186/s13643-023-02338-7 (PMC10500864; doi:10.1186/s13643-023-02338-7)
Supplement: Supplementary file 1 — Additional file 1. Proposed databases, search strategies and results. Medline via EBSCOhost. [file 13643_2023_2338_MOESM1_ESM.docx]

**The perceptions of patients towards the role of community pharmacists in the prevention and control of cardiovascular diseases: a systematic review**

**Additional file 1**

**Proposed Databases, Search Strategies and Results**

***Medline via EBSCOhost***

| **SN** | **SEARCH TERMS** | **RESULTS** |
| --- | --- | --- |
| S1 | Patients OR Clients OR Consumer OR survivor OR user OR customer, people OR persons OR public | 1,535,741 |
| S2 | Perception OR Attitude OR Belief OR Practice OR behaviour OR knowledge OR satisfaction OR awareness OR experiences OR expectation OR willingness OR barriers | 1,177,220 |
| S3 | Community Pharmacist OR Pharmacist | 24,665 |
| S4 | Role OR Contribution OR Interventions OR Services OR Support OR Professional role OR involvement OR engagement, participation OR experiences OR care | 1,771,972 |
| S5 | Prevention OR Control OR Treatment OR management OR primary prevention OR secondary prevention OR diagnosis OR preventive measures | 1,615,542 |
| S6 | Mortality OR Morbidity OR Risk factors OR Incidence OR Prevalence OR Health indicators OR years lived with disability OR disease burden OR deaths OR disability-adjusted life years OR severity OR causes of death OR premature death | 1,187,361 |
| S7 | Cardiovascular disease OR Heart-related disease OR Coronary heart disease OR Cardiovascular risk factors OR cardiovascular events OR cardiovascular health OR atherosclerosis OR coronary artery disease | 236,825 |
| S8 | S1 AND S2 | 289,208 |
| S9 | S3 AND S4 | 5,235 |
| S10 | S5 AND S9 | 3,402 |
| S11 | S6 AND S7 | 43,944 |
| S12 | S10 AND S11 | 108 |
| S13 | S8 AND S12 | 55 |

Search Range: 01/01/2001 - 31/12/2021

***Web of Science***

| **SN** | **SEARCH TERMS** | **RESULTS** |
| --- | --- | --- |
| #1 | **ALL=(Patients OR Clients OR Consumer OR survivor OR user OR customer, people OR persons OR public)** and **Articles** (Document Types) and **English** (Languages) | [5,617,774](https://www-webofscience-com.ukzn.idm.oclc.org/wos/woscc/summary/c89b5d45-7a2b-4599-8c1b-1cabe240a9b0-1e399b37/relevance/1) |
| #2 | **ALL=(Perception OR Attitude OR Belief OR Practice OR behaviour OR knowledge OR satisfaction OR awareness OR experiences OR expectation OR willingness OR barriers)** and **Articles** (Document Types) and **English** (Languages) | [6,712,565](https://www-webofscience-com.ukzn.idm.oclc.org/wos/woscc/summary/b4037cc6-4f0c-4a23-825f-bcc1ca3df193-1e399bc1/relevance/1) |
| #3 | **ALL=(Community Pharmacist OR Pharmacist)** and **Articles** (Document Types) and **English** (Languages) | [27,028](https://www-webofscience-com.ukzn.idm.oclc.org/wos/woscc/summary/51518471-e35d-4d75-8ad3-e8f4c8a7584d-1e399ade/relevance/1) |
| #4 | **ALL=(Role OR Contribution OR Interventions OR Services OR Support OR Professional role OR involvement OR engagement, participation OR experiences OR care)** and **Articles** (Document Types) and **English** (Languages) | [11,844,639](https://www-webofscience-com.ukzn.idm.oclc.org/wos/woscc/summary/4bf199f5-5e5b-4fa7-b70c-88bcb069bde3-1e399a55/relevance/1) |
| #5 | **ALL=(Prevention OR Control OR Treatment OR management OR primary prevention OR secondary prevention OR diagnosis OR preventive measures)** and **Articles** (Document Types) and **English** (Languages) | [9,606,351](https://www-webofscience-com.ukzn.idm.oclc.org/wos/woscc/summary/bbe101a3-3573-4f71-8dd2-92ad8b139d02-1e3999b9/relevance/1) |
| #6 | **ALL=(Mortality OR Morbidity OR Risk factors OR Incidence OR Prevalence OR Health indicators OR years lived with disability OR disease burden OR deaths OR disability-adjusted life years OR severity OR causes of death OR premature death)** and **Articles** (Document Types) and **English** (Languages) | [3,271,733](https://www-webofscience-com.ukzn.idm.oclc.org/wos/woscc/summary/6cbc47b7-3121-4993-83d8-f419cda4fb27-1e39993a/relevance/1) |
| #7 | **ALL=(Cardiovascular disease OR Heart-related disease OR Coronary heart disease OR Cardiovascular risk factors OR cardiovascular events OR cardiovascular health OR atherosclerosis OR coronary artery disease)** and **Articles** (Document Types) and **English** (Languages) | [520,630](https://www-webofscience-com.ukzn.idm.oclc.org/wos/woscc/summary/4aecbc42-95f0-4a8c-b9a2-4a6744bd04a0-1e39974d/relevance/1) |
| #8 | #1 AND #2 | [1,812,018](https://www-webofscience-com.ukzn.idm.oclc.org/wos/woscc/summary/180cccea-8bf0-49bf-9deb-647aaccefd24-1e399cd2/relevance/1) |
| #9 | #3 AND #4 | [20,956](https://www-webofscience-com.ukzn.idm.oclc.org/wos/woscc/summary/fd14d937-5d08-4191-8a7a-c51118bf10f2-1e399d1f/relevance/1) |
| #10 | #5 AND #9 | [12,204](https://www-webofscience-com.ukzn.idm.oclc.org/wos/woscc/summary/5efa11f1-1f9a-4fd1-91c9-e24d35303c1a-1e399d52/relevance/1) |
| #11 | #6 AND #7 | [308,814](https://www-webofscience-com.ukzn.idm.oclc.org/wos/woscc/summary/7b0fef03-aa33-4e89-b724-9d1b1e72419b-1e399dba/relevance/1) |
| #12 | #10 AND #11 | [466](https://www-webofscience-com.ukzn.idm.oclc.org/wos/woscc/summary/f8ef61f2-3ec0-40e5-8555-6e8d3f9174c4-1e399e47/relevance/1) |
| #13 | #8 AND #12 | [281](https://www-webofscience-com.ukzn.idm.oclc.org/wos/woscc/summary/c24b5a32-9f16-47cf-9d04-d0a5b352f7d6-1e399e8b/relevance/1) |

Search Range: 01/01/2001 - 31/12/2021

***CINAHL via EBSCOhost***

Search Range: 01/01/2001 - 31/12/2021

| **SN** | **SEARCH TERMS** | **RESULTS** |
| --- | --- | --- |
| S1 | Patients OR Clients OR Consumer OR survivor OR user OR customer, people OR persons OR public | 14,697 |
| S2 | Perception OR Attitude OR Belief OR Practice OR behaviour OR knowledge OR satisfaction OR awareness OR experiences OR expectation OR willingness OR barriers | 15,585 |
| S3 | Community Pharmacist OR Pharmacist | 351 |
| S4 | Role OR Contribution OR Interventions OR Services OR Support OR Professional role OR involvement OR engagement, participation OR experiences OR care | 15,815 |
| S5 | Prevention OR Control OR Treatment OR management OR primary prevention OR secondary prevention OR diagnosis OR preventive measures | 13,774 |
| S6 | Mortality OR Morbidity OR Risk factors OR Incidence OR Prevalence OR Health indicators OR years lived with disability OR disease burden OR deaths OR disability-adjusted life years OR severity OR causes of death OR premature death | 8,753 |
| S7 | Cardiovascular disease OR Heart-related disease OR Coronary heart disease OR Cardiovascular risk factors OR cardiovascular events OR cardiovascular health OR atherosclerosis OR coronary artery disease | 1,041 |
| S8 | S1 AND S2 | 6,292 |
| S9 | S3 AND S4 | 52 |
| S10 | S5 AND S9 | 22 |
| S11 | S6 AND S7 | 124 |
| S12 | S10 OR S11 | 146 |
| S13 | S8 AND S12 | 60 |
