# Supplementary material for: A systematic review of the role of community pharmacists in the prevention and control of cardiovascular diseases: the perceptions of patients
Source: Syst Rev. 2023 Sep 14;12:160. doi: 10.1186/s13643-023-02338-7 (PMC10500864; doi:10.1186/s13643-023-02338-7)
Supplement: Supplementary file 2 — Additional file 2. [file 13643_2023_2338_MOESM2_ESM.docx]

**Table 2(a):** JBI quality assessment results for qualitative studies

| Quality parameters and scores* | | | | | | | | | | | |
| --- | --- | --- | --- | --- | --- | --- | --- | --- | --- | --- | --- |
| Studies | **#1** | **#2** | **#3** | **#4** | **#5** | **#6** | **#7** | **#8** | **#9** | **#10** | **Total** |
| Al Hamarneh et al (2018). | 0 | 1 | 1 | 1 | 1 | 0 | 1 | 1 | 1 | 1 | 8 |
| Peletidi et al (2019). | 1 | 1 | 1 | 1 | 1 | 1 | 1 | 1 | 1 | 1 | 10 |
| Puspitasari et al (2013). | 1 | 1 | 1 | 1 | 1 | 0 | 0 | 1 | 1 | 1 | 8 |
| **Total** | 2 | 3 | 3 | 3 | 3 | 1 | 2 | 3 | 3 | 3 |  |

*Lockwood et al., 2020

**Table 2(b):** JBI quality assessment results for prevalence studies

| Quality parameters and scores* | | | | | | | | | | |
| --- | --- | --- | --- | --- | --- | --- | --- | --- | --- | --- |
| Studies | **#1** | **#2** | **#3** | **#4** | **#5** | **#6** | **#7** | **#8** | **#9** | **Total** |
| Al Hamarney et al (2012). | 0 | 0 | 0 | 1 | 0 | 1 | 1 | 0 | 0 | 3 |
| Boardman & Avery (2014). | 1 | 0 | 0 | 1 | 0 | 1 | 0 | 1 | 0 | 4 |
| Fonseca et al (2021). | 0 | 0 | 0 | 1 | 0 | 1 | 1 | 0 | 0 | 3 |
| Horgan et al (2010). | 1 | 0 | 0 | 1 | 1 | 1 | 1 | 1 | 0 | 6 |
| Hourihan et al (2003). | 1 | 0 | 0 | 1 | 0 | 1 | 1 | 0 | 0 | 4 |
| Hunt et al (2013). | 1 | 0 | 0 | 1 | 1 | 1 | 1 | 0 | 0 | 5 |
| Jahangard-Rafsanjani et al (2017). | 0 | 0 | 0 | 1 | 1 | 1 | 1 | 0 | 0 | 4 |
| Katoue et al (2013). | 1 | 1 | 1 | 1 | 0 | 1 | 1 | 0 | 1 | 7 |
| Khettar et al (2021). | 0 | 0 | 0 | 1 | 1 | 1 | 1 | 0 | 0 | 4 |
| Kwint et al (2012). | 0 | 0 | 0 | 1 | 1 | 1 | 1 | 1 | 0 | 5 |
| Niquille & Bugnon (2010). | 0 | 0 | 0 | 1 | 1 | 1 | 1 | 1 | 0 | 5 |
| Olenak & Calpin (2010). | 0 | 0 | 0 | 1 | 0 | 0 | 1 | 0 | 0 | 2 |
| Sandhu et al (2018). | 1 | 1 | 0 | 1 | 1 | 1 | 1 | 0 | 0 | 6 |
| Sia et al (2020). | 1 | 0 | 1 | 1 | 1 | 1 | 1 | 0 | 0 | 6 |
| Thompson et al (2020). | 0 | 0 | 0 | 1 | 1 | 1 | 1 | 0 | 0 | 4 |
| van Geffen et al (2011). | 0 | 0 | 0 | 1 | 1 | 1 | 1 | 1 | 0 | 5 |
| **Total** | 7 | 2 | 2 | 16 | 10 | 15 | 15 | 5 | 1 |  |

Munn et al., 2015

**Table 2(c):** JBI quality assessment results for randomised controlled studies and cluster-randomised trials

| Quality parameters and scores* | | | | | | | | | | | | | | |
| --- | --- | --- | --- | --- | --- | --- | --- | --- | --- | --- | --- | --- | --- | --- |
| Studies | **#1** | **#2** | **#3** | **#4** | **#5** | **#6** | **#7** | **#8** | **#9** | **#10** | **#11** | **#12** | **#13** | **Total** |
| Al Hamarney et al 2017 | 0 | 1 | 1 | 0 | 0 | 0 | 1 | 1 | 1 | 0 | 0 | 0 | 0 | 5 |
| Ali et al (2012). | 1 | 1 | 1 | 0 | 0 | 0 | 1 | 1 | 0 | 0 | 0 | 0 | 0 | 5 |
| Jaffray et al (2007). | 1 | 1 | 1 | 0 | 0 | 1 | 1 | 1 | 1 | 1 | 1 | 1 | 1 | 11 |
| Simpson et al (2004). | 0 | 0 | 1 | 0 | 0 | 0 | 1 | 1 | 1 | 1 | 1 | 1 | 1 | 8 |
| Tsuyuki et al (2002). | 1 | 1 | 1 | 0 | 0 | 0 | 1 | 1 | 1 | 1 | 0 | 0 | 1 | 8 |
| Tsuyuki et al (2016). | 1 | 1 | 1 | 0 | 0 | 0 | 1 | 1 | 1 | 0 | 1 | 1 | 1 | 9 |
| Aslani et al (2010). | 0 | 0 | 1 | 0 | 0 | 0 | 1 | 0 | 0 | 1 | 1 | 1 | 1 | 6 |
| Krass et al (2007). | 0 | 0 | 1 | 0 | 0 | 0 | 0 | 1 | 0 | 1 | 1 | 1 | 0 | 5 |
| Okada et al (2016). | 0 | 0 | 1 | 0 | 0 | 0 | 1 | 0 | 0 | 1 | 0 | 1 | 1 | 5 |
| Okada et al (2017). | 1 | 0 | 1 | 0 | 0 | 1 | 0 | 1 | 1 | 0 | 1 | 1 | 0 | 7 |
| Stewart et al (2014). | 1 | 0 | 1 | 0 | 0 | 1 | 1 | 1 | 1 | 1 | 1 | 1 | 1 | 10 |
| Zillich et al (2005). | 1 | 0 | 1 | 0 | 0 | 0 | 1 | 0 | 1 | 1 | 1 | 1 | 0 | 7 |
| Blackburn et al (2016). | 1 | 0 | 1 | 0 | 0 | 0 | 1 | 1 | 0 | 1 | 1 | 1 | 1 | 8 |
| **Total** | 8 | 5 | 13 | 0 | 0 | 3 | 11 | 10 | 8 | 9 | 9 | 10 | 8 |  |

*Tufanaru et al., 2020

**Table 2(d):** JBI quality assessment results for quasi-experimental studies

| **Quality parameters and scores*** | | | | | | | | | | |
| --- | --- | --- | --- | --- | --- | --- | --- | --- | --- | --- |
| Studies | **#1** | **#2** | **#3** | **#4** | **#5** | **#6** | **#7** | **#8** | **#9** | **Total** |
| Aguwa et al (2008). | 1 | 1 | 1 | 0 | 1 | 0 | 1 | 0 | 1 | 6 |
| Al Hamarney et al (2013). | 1 | 1 | 1 | 0 | 1 | 1 | 1 | 1 | 1 | 8 |
| Ali et al (2003). | 1 | 1 | 1 | 0 | 1 | 1 | 1 | 1 | 1 | 8 |
| Cranor et al (2003). | 1 | 1 | 1 | 0 | 1 | 1 | 0 | 1 | 1 | 7 |
| Fahs et al (2018). | 1 | 1 | 1 | 0 | 1 | 1 | 1 | 0 | 1 | 7 |
| John et al (2006). | 1 | 1 | 1 | 0 | 1 | 1 | 1 | 0 | 1 | 7 |
| Mc Namara et al (2015). | 1 | 1 | 1 | 0 | 1 | 0 | 1 | 1 | 0 | 6 |
| Chabot et al (2003). | 1 | 0 | 1 | 1 | 1 | 0 | 1 | 1 | 1 | 7 |
| Oser et al (2017). | 1 | 1 | 1 | 0 | 1 | 0 | 0 | 1 | 1 | 6 |
| Tsuyuki et al (2004). | 1 | 1 | 1 | 0 | 1 | 1 | 1 | 1 | 1 | 8 |
| Marfo & Owusu-Daaku, (2017). | 1 | 1 | 1 | 1 | 1 | 1 | 0 | 1 | 1 | 8 |
| Fikri-Benbrahim et al (2013). | 1 | 1 | 1 | 1 | 1 | 1 | 1 | 1 | 1 | 9 |
| Robinson et al (2010). | 1 | 1 | 1 | 1 | 1 | 1 | 1 | 1 | 1 | 9 |
| **Total** | 13 | 12 | 13 | 4 | 13 | 9 | 10 | 10 | 12 |  |

*Tufanaru et al., 2020
